# Outcomes of non-limited versus cranial-limited extensive hemilaminectomy and durotomy in dogs with thoracolumbar intervertebral disc extrusion and presumptive progressive myelomalacia

**DOI:** 10.1186/s12917-025-04651-w

**Published:** 2025-03-22

**Authors:** Yuya Nakamoto, Kyosuke Hidari, Mei Matsuo, Miwa Nakamoto

**Affiliations:** 1 Neuro Vets Animal Neurology Clinic, 550-4-4F, Bishamon-cho, Nakagyo-ku, Kyoto, 604-0981 Japan; 2 Veterinary Medical Center, School of Veterinary Science, Osaka Metropolitan University, 1-58 Rinku, Ohrai-kita, Izumisano, Osaka 598-8531 Japan

**Keywords:** Canine, MRI, Progressive myelomalacia, Extensive hemilaminectomy and durotomy, Spinal cord, Surgical technique

## Abstract

**Background:**

Progressive myelomalacia (PMM) can occur secondary to thoracolumbar intervertebral disc extrusion (TL-IVDE) and is typically fatal. A recent study suggested that compared with dogs undergoing standard hemilaminectomy, the survival rate of dogs with presumptive PMM improved with non-limited extensive hemilaminectomy and durotomy (EHLD) conducted on the region with a hyperintense intramedullary signal on T2-weighted magnetic resonance imaging (MRI). Applying EHLD results in a large wound and entails prolonged surgical duration. The current study aimed to retrospectively compare the outcomes between an EHLD group and a limited EHLD group (EHLD-L) with TL-IVDE and presumptive PMM. EHLD-L was performed from one additional vertebral body cranial to the spinal cord parenchyma with the T2-weighted hyperintense region identified on MRI, extending caudally until spinal cord swelling and/or softening was visually confirmed during hemilaminectomy and durotomy. Twelve dogs diagnosed with TL-IVDE and presumptive PMM based on clinical features and MRI findings were retrospectively recruited. After diagnosis, seven and five dogs immediately underwent EHLD-L and EHLD, respectively. Medical records of all dogs were retrospectively reviewed, and the postoperative survival outcome, length of the hemilaminectomy window, and surgical operating time between the two groups were compared.

**Results:**

One month after the surgery, the survival rates of the EHLD-L and EHLD groups were 6/7 and 5/5, respectively. The median length of the hemilaminectomy window was 4 (range, 4–6) for the EHLD-L group and 10 (range, 6–14) for the EHLD group (*p* = 0.076). The mean surgical operating times were 106 (range, 80–168) minutes in the EHLD-L group and 192 (range, 128–207) minutes in the EHLD group (*p* = 0.030). There was no improvement in any surviving dogs’ urinary continence or pelvic limb function.

**Conclusions:**

Compared with EHLD, EHLD-L can be associated with a smaller surgical wound and a shorter anesthesia time. EHLD-L did not affect the recovery of pelvic limb function or urinary continence. However, it can be an alternative to EHLD for preserving the life of dogs with presumed PMM.

## Background

Progressive myelomalacia (PMM) is a frequently fatal disorder characterized by progressive ascending and/or descending necrosis of the spinal cord after acute spinal cord injury [1, 2]. PMM can occur secondary to thoracolumbar intervertebral disc extrusion (TL-IVDE) and most often occurs in dogs that have lost deep pain perception in the pelvic limb [1, 3]. The prevalence of PMM with TL-IVDE is approximately 2.0%, ranging from 0 to 14.5% according to clinical grade [4]. In paraplegic dogs with loss of deep pain perception in the pelvic limb, the prevalence of PMM ranges from 10 to 17.5% [2, 4–6]. A study found PMM in 33% of paraplegic French bulldogs without nociception [7]. The clinical signs of PMM include lower motor neuron deficits in the pelvic limb, cranial advancement of the cutaneous trunci reflex, loss of anal and abdominal muscle tone, development of tetraparesis, and/or respiratory paralysis resulting in death [2, 8]. Dogs are usually euthanized before respiratory failure leads to death [1, 2].

Magnetic resonance imaging (MRI) is the preferred method for assessing acute spinal cord injuries in dogs. Several studies have described MRI findings in confirmed cases of PMM, including parenchymal hyperintensity in T2-weighted (T2W) images, likely attributable to cord edema and/or necrosis, as well as diminished parenchymal signals in gradient echo images from hemorrhage [9, 10]. Persistent areas of spinal cord contrast reduction in the subarachnoid space and similar signal loss observed in MRI single-shot turbo spin-echo sequences (such as HASTE) have also been documented, possibly indicating areas of spinal cord swelling [11, 12]. One study highlights an MRI finding suggesting PMM, which is an intramedullary hyperintense signal exceeding six times the length of the second lumbar (L2) vertebral body in T2-weighted (T2W) imaging (T2 length ratio) [9]. A presumptive diagnosis of PMM typically relies on a mix of clinical signs and MRI results.

A recent study suggested that the survival rate of dogs with presumptive PMM improved with extensive hemilaminectomy and durotomy (EHLD) in the region with a hyperintense intramedullary signal on T2W MRI compared with that of dogs who underwent standard hemilaminectomy [13]. In that study, EHLD was performed immediately after the dogs underwent MRI under sustained anesthesia in one session. A similar study in two anesthesia sessions reported that MRI and EHLD were performed at different time points [14]. These reports show that EHLD might effectively inhibits presumptive PMM progression. However, no improvement in the urinary continence or pelvic limb function of any surviving dogs [13, 14]. The EHLD method outlined in previous studies begins with a hemilaminectomy followed by removal of the extruded disc material. The cranial extent of the hemilaminectomy window within the durotomy range was set to one additional vertebral body to the cranial limit of the intramedullary T2W hyperintensity [13]. Therefore, EHLD requires large wounds, and surgery is prolonged [13, 14]. A prior study indicated that a durotomy spanning four vertebral lengths resulted in better functional outcomes for dogs suffering from severe spinal cord injuries following acute TL-IVDH [15]. Therefore, it was deemed feasible that adequate decompression might be accomplished through hemilaminectomy and durotomy conducted within a restricted area. To address the EHLD issue, we devised an EHLD method that can be performed within the cranial-limited section of the T2W MRI hyperintense region of the spinal cord (EHLD-L). The hyperintense region observed on T2W MRI was often not at the site of the extruded disc material; therefore, the extradural material compressing the cord was not removed in the EHLD-L. If patients who underwent EHLD-L versus EHLD had similar outcomes, less invasive procedures, and shorter surgeries could be performed. However, the primary goal of the surgery was to preserve life rather than restore ambulation. The study aimed to compare both techniques and the outcome of dogs with TL-IVDE and presumptive PMM.

## Methods

### Case selection

The medical records of dogs who underwent EHLD-L and EHLD for presumptive PMM from February 2020 to August 2023 at the Neuro Vets Animal Neurology Clinic were collected and evaluated retrospectively. The criteria for inclusion were dogs experiencing acute-onset paraplegia with loss of nociception in the pelvic limbs that underwent a comprehensive diagnostic evaluation, which included physical examinations and a diagnosis of TL-IVDE (with T2 length ratio ≥ 6) verified through MRI. For the deep pain assessment, the digits of both pelvic limbs and the tail were clamped with forceps. Deep pain presence or absence was determined by a distinct and reproducible behavioral response to noxious stimuli interpreted as pain (e.g., sudden head-turning, attempting to bite the source of the stimulus). The EHLD-L and EHLD groups were treated surgically immediately after MRI in one general anesthesia session. The same single surgeon performed EHLD-L and EHLD. The cranial site for EHLD-L was defined as the most cranial region of the spinal cord parenchyma with T2W MRI hyperintensity. The EHLD-L and EHLD techniques were explained to the owner, who selected the procedure performed. The minimum follow-up period of the two groups was 6 months or until death or euthanasia.

Data were assessed from the medical records and preoperative examinations, including the signalment, T2 length ratio, and surgery type. Informed consent for EHLD-L and EHLD and information collection for research purposes was obtained from the owners of all dogs.

All dogs received anesthesia and had MRIs (0.4 T, Hitachi APERTO Lucent; Hitachi Medical Systems, Tokyo, Japan) of the thoracolumbar vertebral column, with protocols including sagittal and transverse T2-weighted images. Intramedullary T2W hyperintensity was present in the sagittal section on MRI. Open-source medical imaging software (OsiriX, www.osirix-viewer.com) was used to calculate the T2 length ratio, as described in previous studies [9, 13, 16]. The surgical operating time was defined as the time from incision to final suture. The study was approved by the Ethics Committee of Neuro Vets Animal Neurology Clinic (Approval number: NVANC-5). ARRIVE guidelines were followed for this study.

#### Preoperative preparation and anesthesia

The dogs received intravenous (IV) propofol (6 mg/kg), and anesthesia was maintained with isoflurane (2–3% in 100% oxygen). Before surgery, the dogs received 22 mg/kg of cefazolin sodium, 2 mg/kg of ranitidine via IV and 0.02 mg/kg of buprenorphine subcutaneously. All dogs received IV fluids during surgery.

#### The surgical method in EHLD-L

**Fig. 1:**
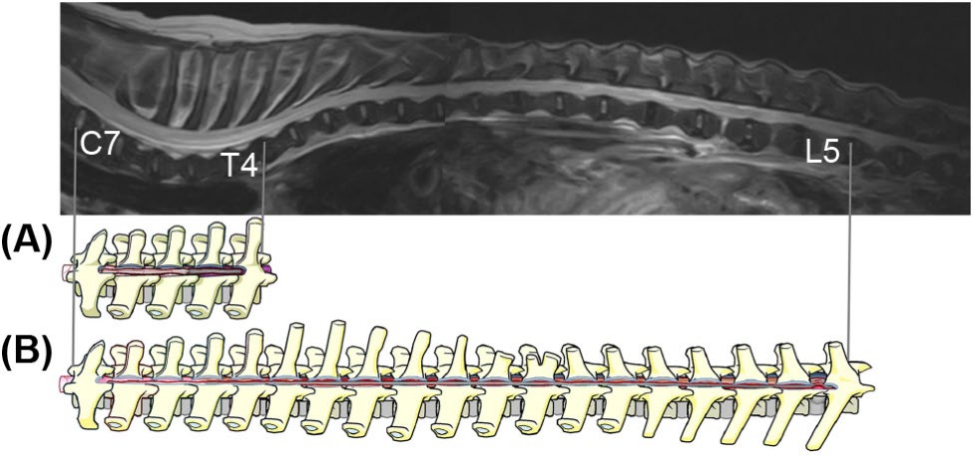
Schematic depiction comparing EHLD-L with that after EHLD performed hypothetically in the same dog. Surgical image of an EHLD-L method in a dog (no. 5). (**A**) EHLD-L was performed from the site of the cranial region with intramedullary hyperintensity on T2W-MRI toward the caudal site until the spinal cord with swelling or softening was visible (C7–T4). (**B**) If EHLD, as previously reported, is applied, spinal decompression from C7 to L5 is required

The dogs were placed in the sternal recumbent position. First, the cranial extent of the hemilaminectomy with durotomy range was set to one additional vertebral body to the cranial side of the T2W hyperintense region as identified on MRI and extended caudally until spinal cord swelling or softening was intraoperatively confirmed by visual inspection (Fig. 1). To attempt to prevent the development of functional abnormalities in the forelimb, hemilaminectomy was extended caudally beyond the second thoracic vertebrae if an abnormal signal intensity was observed in the sixth cervical vertebrae to the second thoracic vertebrae region. Routine closure of the surgical wound was performed. The TL-IVDE site was not included in EHLD-L.

#### Surgical method in EHLD

The dogs were placed in sternal recumbency. EHLD was performed as described in a previous study [13].

### Perioperative care

The dogs received prednisolone (0.5–1.0 mg/kg/day subcutaneously for 2 days), cefazolin sodium (22 mg/kg twice per day via IV or subcutaneously until discharge), and ranitidine (2 mg/kg twice per day via IV or subcutaneously until discharge). A constant-rate infusion of morphine (0.1–0.2 mg/kg/h, IV) was administered for the initial 48 h after surgery, followed by buprenorphine (0.02 mg/kg subcutaneously twice daily until discharge) after the morphine regimen CRI. The minimum length of hospital stay after the surgery was 5 days. The patients were deemed ready for discharge if they were in good health and had no respiratory abnormalities or worsening clinical signs related to PMM. After discharge from the hospital, oral cephalexin (22 mg/kg twice daily) and tramadol (5 mg/kg twice daily) were administered for 7–10 days. The postoperative care instructions provided to the owners covered urinary management (such as manual bladder expression), skin care (like treating dermatitis), physiotherapy (including passive range of motion exercises and massage), and wheelchair use.

### Postoperative follow-ups

The dogs were monitored by performing follow-up examinations 10–14 days after discharge and then by the authors or referring veterinarians monthly for 2–4 months. The dogs’ general health and neurological signs were evaluated during those examinations. Thereafter, regular follow-ups were performed as telephone consultations.

### Statistical analysis

R software, version 4.3.1 (R Foundation for Statistical Computing), was used to perform statistical analyses. The age, sex, T2 length ratio, length of the hemilaminectomy window, surgical operating time, and survival outcome were compared between the two groups. In both analyses, Wilcoxon rank sum tests and Fisher’s exact test were applied to categorical and continuous variables, irrespective of data distribution, due to the limited sample size sizes. Statistical significance was set at *p* < 0.05.

## Results

### Study population

Twelve dogs (seven with EHLD-L and five with EHLD) were included in the study (Table 1). The dog breeds in the EHLD-L group comprised Miniature Dachshunds (*n* = 3), a French Bulldog (*n* = 1), a Toy Poodle (*n* = 1), an American Cocker Spaniel (*n* = 1), and a mixed breed (*n* = 1). The dog breeds in the EHLD group comprised Miniature Dachshunds (*n* = 3) and Toy Poodles (*n* = 2). At presentation, the median ages of the dogs were 50 (interquartile range (IQR): 33–63) months in the EHLD-L group and 67 (IQR: 46–73) months in the EHLD group (Table 1). There were 11 castrated male dogs and 1 spayed female dog. The median number of days from onset to presentation at the Neuro Vets Animal Neurology Clinic was 4 (IQR, 3–7) days in the EHLD-L group and 4 (IQR, 2.5–4.5) days in the EHLD group (Tables 1 and 2). No differences were observed regarding age, sex, or the time from onset to presentation (Table 2). Neurological assessments at the time of presentation indicated upper motor neuron signs—such as heightened spinal reflexes—in the pelvic limbs of 3 dogs in the EHLD-L group and 4 dogs in the EHLD group. Furthermore, lower motor neuron signs (for instance, reduced or absent spinal reflexes) in the pelvic limbs were observed in 4 dogs from the EHLD-L group and 1 dog from the EHLD group.

**Table 1:** Signalment of dogs with presumptive PMM

Dog no.	Breed	Age, mo	Sex	Interval between onset and presentation, d	IVDE site	Range of EHLD-L and EHLD	Cranial and caudal extent of lesion	T2 length ratio	Procedure	Neurological findings of pelvic limbs			Length of hospital stay, d	Surgical operating time, min	Survival outcome	Follow-up, d
										At the time of presentation		At the time of discharge				
1	TP	43	M/C	4	L4-5	T3-8	T4-L7	17.2	EHLD-L	LMNs	LMNs		6	106	Alive	725
2	MD	26	M/C	4	T11-12	T2-6	T3-L5	18.9	EHLD-L	UMNs	LMNs		5	80	Alive	601
3	FB	176	M/C	7	L1-2	C7-T4	T1-L7	20.6	EHLD-L	LMNs	LMNs		7	96	Alive	329
4	Mixed	63	M/C	8	L1-2	C7-T6	T1-L7	21.7	EHLD-L	LMNs	LMNs		6	168	Alive	301
5	MD	50	M/C	3	L4-5	C7-T4	T1-L7	23.3	EHLD-L	LMNs	LMNs		6	137	Alive	230
6	MD	50	M/C	3	L2-3	T1-6	T2-L4	15.8	EHLD-L	UMNs	LMNs		7	102	Alive	444
7	AC	33	M/C	5	T12-13	C7-T4	C7-L3	16.9	EHLD-L	UMNs	LMNs		7	108	Dead (due to bladder bleeding and lacerations)	13
8	TP	71	M/C	2	L1-2	T5-L2	T6-L2	13.8	EHLD	UMNs	UMNs		7	192	Alive	1580
9	MD	75	F/S	4	T13-L1	C7-L1	T1-L3	19.2	EHLD	UMNs	UMNs		7	207	Alive	1185
10	MD	67	M/C	4	L1-2	T9-L2	T10-L2	10.8	EHLD	UMNs	LMNs		6	131	Alive	755
11	TP	48	M/C	3	L3-4	T11-L4	T12-L4	10.9	EHLD	UMNs	LMNs		5	128	Alive	665
12	MD	44	M/C	5	L2-3	T3-L3	T4-L3	18.3	EHLD	LMNs	LMNs		6	209	Alive	618

*Abbreviations*: AC, American Cocker Spaniel; C, cervical; d, days; EHLD, non-limited extensive hemilaminectomy and durotomy; EHLD-L, extensive hemilaminectomy and durotomy within the cranial-limited region of the spinal cord; FB, French Bulldog; F/S, female spayed; IVDE, intervertebral disc extrusion; L, lumbar; LMNs, lower motor neuron sign; MD, Miniature Dachshund; Mixed, mixed breed; M/C, male castrated; min, minute; mo, month; PMM, progressive myelomalacia; T, thoracic; TP, Toy Poodle; UMNs, upper motor neuron sign; y, years

**Table 2:** Characteristics of the study population

Variables	EHLD-L group, *n* = 7	EHLD group, *n* = 5	*p*-value
Age at presentation, median (IQR), months	50 (33–63)	67 (46–73)	0.33 (a)
Sex, n			0.42 (b)
M/C	7	4	
F/S	0	1	
Days from onset to presentation, median (IQR)	4 (3–7)	4 (2.5–4.5)	0.36 (a)
T2 length ratio, median (IQR)	18.9 (16.9–21.7)	13.8 (10.85–18.75)	0.11 (a)
Length of the hemilaminectomy window, median (IQR)	4 (4–5)	10 (6.5–13.5)	0.0076 (a)
Surgical operating time, median (IQR), minutes	106 (96–137)	192 (129.5–208)	0.030 (a)
Postoperative survival, n	6/7	5/5	1.0 (b)
Alive	6	5	
Dead	1	0	

*Abbreviations*: EHLD, non-limited extensive hemilaminectomy, and durotomy; EHLD-L, extensive hemilaminectomy, and durotomy within the cranial-limited region of the spinal cord; F/S, female spayed; M/C, male castrated; SD, standard deviation

IQR, Interquartile (25th–75th percentile) range

(a) Wilcoxon rank sum exact test. (b) Fisher’s exact test

### MRI findings

The distributions of the T-L IVDE site were T11–12 (*n* = 1), T12–13 (*n* = 1), T13–L1 (*n* = 1), L1–2 (*n* = 4), L2–3 (*n* = 2), L3–4 (*n* = 1), and L4–5 (*n* = 2). The median T2 length ratios were 18.9 (IQR, 16.9–21.7) in the EHLD-L group and 13.8 (IQR, 10.85–18.75) in the EHLD group (Table 1). There was no significant difference in the T2 length ratio between the two groups (*p* = 0.11, Wilcoxon rank sum exact test) (Table 2).

### Surgical details

The median length of the hemilaminectomy window of the EHLD-L group was 4 (IQR, 4–5), and the EHLD group was 10 (range, 6.5–13.5) (Table 1). The length of the hemilaminectomy window was significantly shorter in the EHLD-L group than in the EHLD group (*p* = 0.0076, Wilcoxon rank sum exact test) (Table 2). In the EHLD-L group, five dogs had liquefied spinal cord tissue that could not maintain its shape, i.e., malacia of the spinal cord, which was diagnosed grossly within the hemilaminectomy and durotomy window at the time of surgery. In the EHLD group, all dogs had malacia of the spinal cord, which was also diagnosed grossly within the hemilaminectomy and durotomy window at the time of surgery. No significant intraoperative complications requiring therapeutic interventions occurred. The median surgical operating time was 106 (IQR, 96–137) minutes in the EHLD-L group and 192 (IQR, 129.5–208) minutes in the EHLD group (Table 1). The surgical time was significantly shorter in the EHLD-L group than in the EHLD group (*p* = 0.030, Wilcoxon rank sum exact test) (Table 2).

### Perioperative outcomes

The dogs were discharged from the hospital a median of 6 (range, 5–7) days after surgery. On neurological examination at discharge, upper motor neuron signs were recognized in two dogs from the EHLD group. In comparison, lower motor neuron signs were recognized in seven dogs from the EHLD-L group and three dogs from the EHLD group. Two weeks after surgery, the perioperative survival was 6/7 in the EHLD-L group and 5/5 in the EHLD group (Table 1). One dog (dog 7) in the EHLD-L group died 13 days after surgery at the office of the referring physician due to hematuria and reduced vitality following hospital discharge; abdominal ultrasound revealed bladder laceration as the cause of death.

### Postoperative outcomes and follow-up

No dogs were lost to follow-up. The median length of follow-up was 386.5 (IQR, 301–601) days in the EHLD-L group and 755 (IQR, 641.5–1382.5) days in the EHLD group. There was no significant difference in the survival outcome values between the two groups (*p* = 1.0, Fisher’s exact test) (Table 2). The surviving dogs remained alive throughout the follow-up period, and none experienced any serious postoperative complications. No neurological abnormalities affecting the thoracic limbs were observed. At the time of the last telephone consultations with the owners during the follow-up period, no improvement based on assessment of pelvic limb function was reported in any of the patients, and there was no functional recovery of urinary or fecal continence.

## Discussion

The study results suggest that EHLD-L has the potential to be a life-saving treatment for dogs with TL-IVDE and presumptive PMM. We found no significant difference in the survival between the EHLD-L and EHLD groups. Previous studies on EHLD have reported survival rates of 31/34 and 10/10 [13, 14]. In the EHLD-L group, the 6/7 dogs had survived 2 weeks after surgery. Furthermore, no dog died during the follow-up period thereafter. The pathology of PMM involves elevated intramedullary pressure [8, 17, 18]. Recent studies suggest that spinal decompression with EHLD inhibits further progression of PMM, even in cases with a presumptive diagnosis of advanced PMM [13, 14]. In this study, three dogs in the EHLD-L group and two in the EHLD group exhibited upper motor neuron (UMN) signs in their pelvic limbs at presentation, transitioning to lower motor neuron (LMN) signs by discharge. This finding indicates that PMM advanced caudally in these cases; however, the durotomy may effectively inhibit ascending myelomalacia. Consequently, the study suggests spinal decompression using EHLD-L and EHLD can halt suspected PMM’s progression. Although EHLD does not aim to restore pelvic limb function, it can be a life-saving intervention for PMM [13, 14]. By preventing PMM progression, EHLD-L and EHLD aim to preserve thoracic limb and respiratory muscle function. According to the literature, the majority of dogs with presumptive and confirmed PMM are euthanized within 4 days following onset, although delayed progression to euthanasia may take as long as 2 weeks [1]. The present study included cases in which EHLD-L was performed 7–8 days after onset, but it is possible that these were cases with slow PMM progression. One dog (dog 7) died due to bladder bleeding and bladder lacerations after being discharged from the hospital. There was no evidence of progressive PMM at the time of death in this patient. In animals with severe spinal cord disorders related to IVDE and other conditions, nursing care for urinary issues and dermatitis is critical [19–21]. Thus, EHLD-L or EHLD is only likely relevant for owners who are comfortable managing a permanently paraplegic and incontinent dog. Otherwise, humane euthanasia can be considered. EHLD-L and EHLD can be useful treatment options because Japanese people are less likely to elect euthanasia due to their national characteristics. However, it is common for dog owners in Europe and the U.S to select euthanasia if their dogs that develop PMM. Nevertheless, this is an ethical dilemma.

A diagnosis of presumptive PMM was made based on MRI features in the present study. Hyperintensity on T2W MRI is a nonspecific finding and may occur in dogs with spinal cord edema, hemorrhage, inflammation, gliosis, necrosis, or myelomalacia [9, 16, 22]. Softening and liquefaction of the spinal cord were seen at the intervertebral disc herniation (IVDH) site and neighboring areas with T2W hyperintensity [13–15, 23, 24]. There is a mixture of softened and enlarged regions extending cranially and caudally from the site of spinal cord damage, such as the IVDH site. The most cephalic region with T2W hyperintensity presents with changes involving edema in the spinal cord [13, 14, 17]. This study involved performing EHLD-L from the cranial region, where intramedullary hyperintensity was observed on T2W-MRI, toward the caudal site, reaching areas of the spinal cord with softening and/or enlargement. The median length of the hemilaminectomy vertebrae in the EHLD-L group was 4 windows. A previous study indicated that a durotomy extending for 4 vertebral lengths enhanced functional outcomes in dogs suffering from severe spinal cord injuries following acute TL-IVDH [15]. This finding indicates that spinal cord decompression can be achieved within the hemilaminectomy window. In another study, approximately 22.6% of patients with a T2 length ratio of 6 or greater experienced improved pelvic limb function following the local hemilaminectomy and durotomy using four windows [23]. However, none of the surviving dogs in the EHLD-L group regained their pelvic limb function.

Reportedly, extended areas of intramedullary T2W hyperintensity indicated poor long-term prognosis [3, 9, 22], whereas another study found that the length of intramedullary T2W hyperintensity did not significantly differ between the dogs that experienced early motor function recovery and those that did not [25]. In dogs with PMM, T2-weighted images of the spinal cord reveal hyperintensity caused by irreversible, progressive necrosis of the parenchyma, accompanied by or without intradural hemorrhage [9]. Reportedly, extensive areas of intramedullary T2W hyperintensity strongly correlated with clinically evident PMM [26]. In one report, a suggestive MRI finding indicative of PMM is a T2 length ratio of 6 or greater [9]. Some lengthy regions in the subarachnoid space exhibit signal loss on HASTE, which may indicate PMM [11, 12]. Nevertheless, one study found the positive predictive values for the T2 length ratio and HASTE signal loss to be 65% and 62%, respectively. In contrast, the absence of these signals had negative predictive values of 81% and 77% [26]. Furthermore, a high-field MRI study demonstrated that dogs with a T2 length ratio of ≥ 6 can survive [25]. This study featured cases that might have survived without the performance of EHLD-L, indicating a need for further thought on the criteria for selecting appropriate candidates.

The rationale for adding durotomy to surgical decompression is that durotomy may improve spinal cord blood flow, is safe, and may result in better functional recovery than laminectomy alone in dogs with spinal cord injury [27]. A recent study prospectively examined a cohort of 26 paraplegic dogs who underwent laminectomy and durotomy for deep pain perception loss secondary to TL-IVDE: 16 of 22 dogs (72%) regained independent ambulation, and only 1 dog (5%) developed PMM [23]. Additionally, durotomy for dogs with severe neurological signs may improve outcomes and lessen the risk of PMM according to some studies [13, 23]. However, recent studies with large sample sizes have reported no significant therapeutic effect of durotomy [24]. Thus, it might not be appropriate to perform durotomy to restore function. Durotomy can also cause spinal herniation, meningeal scarring, and adhesion formation [27]. Although these findings have not been reported in the literature on EHLD, laminectomy membrane formation can be a late complication. Consequently, keeping the extent of durotomy as small as possible may be better.

EHLD is highly invasive to the spinal cord and muscles due to the larger surgical wound. Thus, the risk of postoperative complications could be high. However, previous studies did not report major complications after EHLD [13, 14]. Additionally, one study reported that multiple hemilaminectomies did not decrease the stiffness of the lumbar spinal column during flexion and extension [28]. Hence, the probability of vertebral instability occurring in EHLD or EHLD-L is low. Moreover, a smaller surgical wound prevents unnecessary muscle damage, shortens operative time, and reduces blood-loss volume and infection risks based on previous studies [29–33]. In addition, one study reported that longer surgical operating and anesthesia times are a possible negative prognostic factor [34]. These findings suggest that a smaller surgical wound can reduce surgical operating time and anesthesia time, decreasing the burden on dogs. In this study, the surgical operating time was shorter in the EHLD-L group than in the EHLD group. Further, there was less extensive hemilaminectomy in EHLD-L than in EHLD. Therefore, the EHLD-L technique can reduce the physical burden on dogs. However, the dog’s breed, size, weight, and other factors that may affect surgical time are also considered. As this study did not examine these factors, further research is needed. Our study did not identify any major complications caused by EHLD-L. Nonetheless, monitoring for potential postoperative adverse events after prolonged surgery is necessary.

Prednisolone, a glucocorticoid, was used in this study. Glucocorticoids may protect the injured spinal cord [35, 36]. Glucocorticoids are also often used during neurosurgery in veterinary medicine [37]. A retrospective study assessed clinical risk factors linked to PMM in dogs with loss of nociception and indicated that corticosteroid treatment might offer a protective effect [1]. Nevertheless, no proof exists that glucocorticoids effectively enhance motor function [19, 27]. Additionally, glucocorticoids can lead to side effects impacting all major organ systems, including musculoskeletal, gastrointestinal, cardiovascular, endocrine, neuropsychiatric, dermatologic, ocular, and immunologic effects in veterinary and human medicine [27, 38]. Corticosteroid usage is increasingly discouraged in acute spinal cord injury cases within both veterinary and human medicine, though a definitive conclusion has not been achieved [27, 39]. As a result, the administration of glucocorticoids is still debated.

In this study, antibiotics were administered until suture removal to prevent surgical wound infections and urinary tract infections. However, in several cases, long-term antibiotic therapy after surgery is not recommended [40] because there is no clear evidence showing the efficacy of long-term postoperative antibiotic therapy for preventing infection. Therefore, long-term postoperative antibiotic administration may not be implemented in EHLD-L and EHLD. However, future studies on surgical wound size and duration of antibiotic administration must be performed.

The current study has several limitations. First, the study was not randomized; therefore, selection bias could have occurred when performing the EHLD-L or non-limited EHLD surgical method. Second, the lack of clinical examination features is consistent with PMM as an inclusion criterion. It is undeniable that dependence on MRI can result in possible misdiagnosis. Furthermore, spinal cord softening relied on gross observation, while histopathological evaluation was not performed. Third, the study sample size was small, meaning the results might not be similar to those in a larger, more diverse sample. For this reason, there was a substantial risk of a type 2 error. Finally, we used low-field MRI; therefore, it may include cases not suggested as PMM if a high-field MRI had been used.

## Conclusions

Compared with non-limited EHLD, EHLD-L can be associated with a smaller surgical wound and shorter anesthesia time. EHLD-L did not affect the recovery of pelvic limb function or urinary continence. Nevertheless, our results support using EHLD-L as an alternative to non-limited EHLD for preserving the life of dogs with presumed PMM.

## Data Availability

The raw data supporting the conclusions of this article will be made available by the authors, without undue reservation.

## Abbreviations

EHLD: Extensive hemilaminectomy and durotomy
EHLD-L: Cranial-limited extensive hemilaminectomy and durotomy
IVDE: Intervertebral disc herniation
MRI: Magnetic resonance imaging
PMM: Progressive myelomalacia
T2W: T2-weighted
